# Dietary Nucleotides Retard Oxidative Stress-Induced Senescence of Human Umbilical Vein Endothelial Cells

**DOI:** 10.3390/nu13093279

**Published:** 2021-09-20

**Authors:** Na Zhu, Xinran Liu, Meihong Xu, Yong Li

**Affiliations:** Department of Nutrition and Food Hygiene, School of Public Health, Peking University, Beijing 100191, China; summer920503@163.com (N.Z.); liuhappy07@163.com (X.L.); xumeihong@bjmu.edu.cn (M.X.)

**Keywords:** dietary nucleotides, senescence, HUVECs, oxidative stress, inflammation, mitochondrial function

## Abstract

Several lines of evidence suggest an inhibitory role of dietary nucleotides (NTs) against oxidative stress and inflammation, which promote senescence in age-associated cardiovascular diseases. We sought to test whether the dietary NTs could retard the hydrogen peroxide (H_2_O_2_)-induced senescence of human umbilical vein endothelial cells (HUVECs) and to elucidate the efficiency of different NTs as well as the potential mechanism. Senescence was induced in HUVECs by 4 h exposure to 200 µM H_2_O_2_ and was confirmed using senescence-associated-β-galactosidase staining (SA-β-gal), cell viability, and Western blot analyses of p16^INK4A^ and p21^Waf1/Cip1^ after 24 h administration of growth medium. We find that NTs retards oxidative stress-induced HUVECs senescence, as shown by a lower percentage of SA-β-gal-positive cells, lower expression of p16^INK4A^, and p21^Waf1/Cip1^ as well as higher cell viability. GMP100 was the most excellent in delaying HUVECs senescence, which was followed by the NTs mixture, NMN, CMP50, and UMP50/100, while AMP retards HUVECs senescence by specifically reducing p15^INK4b^ expression. NTs all have significant anti-inflammatory effects; AMP and CMP were more prominent in restoring mitochondrial function, GMP and CMP were more competent at eliminating ROS and MDA, while AMP and UMP were more efficient at enhancing antioxidant enzyme activity. The role of the NTs mixture in retarding HUVECs senescence is full-scaled. These results stated that the mechanisms of NTs retarding HUVECs senescence could be related to its antioxidant and anti-inflammation properties promoting cell proliferation and protecting mitochondrial function activities.

## 1. Introduction

Cardiovascular diseases (CVDs) are the leading cause of death globally [1], and the strongest independent risk factor for CVDs is age: more than 90% of CVDs occur in adults age 40 and older [2]. As a hallmark of aging, senescent cells could play a detrimental role in age-associated pathologies [3,4]. Cellular senescence is a stable cell cycle arrest [5] that is characterized by an inflammatory phenotype known as the senescence-associated secretory phenotype (SASP) [6], the accumulation of oxidative stress-induced damage [7,8], telomere shortening [9], and mitochondrial dysfunction [10]. The accumulation of senescent cells with age might trigger a chronic inflammation with detrimental effects on neighboring cells and the whole organism, therefore contributing to the initiation and progression of CVDs [11]. Indeed, there is a large body of evidence that senescent cells are present in the pathological tissues of patients with CVDs [12]. The expression of senescence markers including SA-β-gal, p16^INK4A^, and p21^Waf1/Cip1^ was upregulated in endothelial cells from human atherosclerotic plaques [13]. Nevertheless, a treatment that attempts to eliminate p16-positive senescent cells could be used to prevent and treat the CVDs [14,15,16].

The data from experimental studies suggest that NTs may have the potential to ameliorate cell senescence. NTs are the basic units of nucleic acid macromolecules, including cytosine, adenine, guanine, thymine, and uracil. These are a group of bioactive agents important to physiological and biochemical functions in organisms; thus, they are self-synthesized by organisms. Meanwhile, NTs absorbed and utilized from the diet are indispensable under certain physiological conditions, such as intestinal injury, immune challenge, starvation, and rapid growth [17,18]. Researchers have reported an increasing number of biological functions of exogenous NTs. Extensive literature links NTs with cell proliferation, demonstrating that NTs could promote human adipose-derived stem cells [19], hepatocytes [20,21], intestinal cells [22], and lymphocytes [23] proliferation. The anti-oxidative effect of NTs has been assessed in several studies, and all of them demonstrated that NTs exhibit superior antioxidant properties [24,25]. NTs have been shown to modulate the immune response, positively influencing immunity, tissue growth, development, and repair [26,27]. Additionally, earlier studies indicate that NTs are responsible for the restoration of mitochondrial function and augmentation of mitochondrial ATPase, citrate synthase, and malate dehydrogenase activities after chronic stress [20,28]. Those facts above may allow us to suppose that NTs may exert senescence ameliorative effect on HUVECs. However, no available studies explored the senescence ameliorative effect of NTs. Therefore, the present study was performed to investigate the possible senescence ameliorative effect of NTs against H_2_O_2_-induced premature senescence in HUVECs and the underlying mechanism.

## 2. Materials and Methods

### 2.1. Chemicals

The NTs used in our experiment are 5′-guanosine monophosphate disodium salt (GMP), 5′-disodium uridine-5′-monophosphate (UMP), 5′-cytimidine monophosphate (CMP), 5′-adenosine monophosphate (AMP), and nicotinamide mononucleotide (NMN), respectively. All of them were supplied by HAINAN SHUANGDI ZHEN-AO LIFE SCIENCE RESEARCH CENTER Co. Ltd. (Baoting, China).

### 2.2. Cell Cuture and Teatments

HUVECs were obtained from American Type Culture Collection (ATCC, Manassas, VA, USA). HUVECs was cultured in Dulbecco’s Modification of Eagle’s Medium (DMEM) (GIBCO, Grand Island, NE, USA) supplemented with 10% fetal bovine serum (Zhong Qiao Xin Zhou Biotechnology Co. Ltd., Shanghai, China) and 1% antibiotic–antimitotic (Coolaber, Beijing, China) at 37 °C under a humidified atmosphere of 5% CO_2_.

The induction of HUVECs senescence by H_2_O_2_ was performed as previously described; HUVECs were maintained for 4 h in a growth medium containing 200 µM H_2_O_2_ (determined by preliminary tests, Figure S1) and then cultured in growth medium with or without different concentrations of NTs for 24 h. Our experiment set up 16 groups in total: a control group (growth medium), model group (growth medium), NTs mixture group (growth medium containing 100 µM NTs mixture, AMP:CMP:GMP:UMP = 22.80:25.80:30.20:20.40), NMN group (growth medium containing 0.5 mM NMN), and low, middle, and high doses of GMP/UMP/CMP/AMP groups (growth medium containing 50/100/200 µM GMP/UMP/CMP/AMP). Following exposure to NTs, the cells were harvested for further analysis.

### 2.3. Morphology Observation

The cell morphological changes were observed by transmission electron microscope. In brief, cells were collected and washed twice with PBS before being fixed with 2.5% glutaraldehyde overnight at 4 °C. Then, cells were washed three times with PBS and fixed with 1% osmic acid for 1 h. Graded acetone was dehydrated (30%, 70%, 95%, 100% in PBS, each for 15 min), the resin was embedded, and ultrathin sections (Mode: OMU3, Leica Reichert, Munich, Germany) were stained with uranyl acetate and citric acid (Beyotime, Shanghai, China). Cellular morphology and mitochondria were observed by transmission electron microscopy (JEM-1400. Leica Reichert, Wetzlar, Germany).

### 2.4. Cell Viability Assay

Cell viability was evaluated by the cell-counting kit-8 (CCK-8) assay (KeyGEN, Jiangsu, China) according to the manufacturer’s protocol. In brief, 100 µL/well cells (about 1 × 10^4^) were seeded in 96-well plates. After treatment according to the protocol, 10 µL CCK-8 was added to each well and incubated at 37 °C for 1–4 h. The absorbance of each well was measured at 450 nm with a microplate reader (BMG FLUOstar Omega, Offenburg, Germany).

### 2.5. Senescence-Associated Beta-Galactosidase (SA-β-gal) Activity

The SA-β-Gal activity was assayed by the SA-β-gal staining kit (Beyotime, Shanghai, China) following the manufacturer’s instructions. In brief, 1 mL/well cells (about 1 × 10^5^) were seeded in 24-well plates. After treatment according to the protocol, the cells were washed once with PBS and fixed with SA-β-gal staining stationary liquid for 15 min at room temperature. After removing the stationary liquid, the cells were washed three times with PBS. Then, cells were incubated overnight at 37 °C in darkness with the SA-β-gal staining working solution. After being rinsed with PBS, the cells were observed at the microscope for the development of the blue coloration. The percentage of SA-gal-positive cells was calculated from three random fields by ImageJ software.

### 2.6. Flow Cytometry

First, 2 mL/well cells (about 2 × 10^5^) were seeded in 6-well plates and treated according to the protocol. For apoptosis analysis, cells were harvested and washed once with PBS and then resuspended in PI/Annexin-V solution (KeyGEN, Jiangsu, China) and analyzed using a Flow Cytometer (Beckman Coulter, Brea, CA, USA). For intracellular ROS analysis, cells were harvested and washed once with PBS before being incubated for 20 min at 37 °C with the 10 µM 2,7-dichlorofluorescein diacetate (Beyotime, Shanghai, China). After being washed with PBS three times, the cells were analyzed using a Flow Cytometer (Beckman Coulter, Brea, CA, USA). For mitochondrial membrane potential (∆Ψm) analysis, cells were harvested and stained with 500 µL 1× JC-1 dye solution (Beyotime, Shanghai, China) at 37 °C for 20 min in the dark. Then, the cells were washed twice and resuspended by 1× JC-1 staining buffer. The change of fluorescence color was analyzed using flow cytometry (Beckman Coulter, Brea, CA, USA).

### 2.7. Biochemical Analysis

First, 2 mL/well cells (about 2 × 10^5^) were seeded in 6-well plates and treated according to the protocol. Then, the supernatant was obtained for the measurements of malondialdehyde (MDA), glutathione peroxidase (GSH-Px), superoxide dismutase (SOD) activities, NAD^+^/NADH, ATP, interleukin-6 (IL-6), IL-1β, IL-17, matrix metalloproteinase-3 (MMP-3), intercellular cell adhesion molecule-1 (ICAM-1), and vascular cell adhesion molecule-1 (VCAM-1) using commercial kits.

### 2.8. Western Blot Analysis

First, 2 mL/well cells (about 2 × 10^5^) were seeded in 6-well plates and treated according to the protocol. Cells were collected and washed twice with PBS; then, they were resuspended in RIPA Lysis Buffer (Biosharp, HeFei, China) and supplemented with 1 mM of phenylmethanesulfonyl fluoride. Protein was extracted by centrifugation at 14,000× *g* for 15 min at 4 °C, and the concentration of protein was measured with a BCA protein assay kit (Thermo Scientific, Waltham, MA, USA). Equal amounts of protein (80–150 µg) were separated by 10–20% SDS-PAGE gel and transferred to PVDF membranes at different electric currents according to the size of protein molecules. The membranes were blocked for 2 h in 5% non-fat milk dissolved with Tris-buffered saline containing 0.05% Tween-20 (TBST) at room temperature. Protein expression was detected using a primary antibody p16^INK4A^ (1:1000, CST, Danvers, MA, USA), p21^Waf1/Cip1^ (1:1000, CST, Danvers, MA, USA), p15^INK4b^ (1:500, Abcam, Cambridge, MA, USA), ACE-2 (1:1000, CST, Danvers, MA, USA), β-actin (1:5000, Abcam, Cambridge, MA, USA), and horseradish peroxidase-conjugated anti-rabbit secondary antibodies (1:10,000, Abcam, Cambridge, MA, USA). Quantitative analysis of Western blot was performed using Image-Pro Plus (Media Cybernetics, Rockville, MD, USA).

### 2.9. Statistical Analysis

Statistical analyses were performed using the SPSS software version 24 (SPSS Inc., Chicago, IL, USA). Data were expressed as mean ± standard deviation (SD) and analyzed by one-way analysis of variance (ANOVA) test; the difference of parametric samples among groups multiple comparisons of least significant difference (equal variances assumed) or Dunnett’s T3 test (equal variances not assumed) was used. *p* < 0.05 indicated a statistically significant difference. The calculating formula of the relative rate is as follows: decreased rate = (model group − treated group)/model group, increased rate = (treated group − model group)/model group.

## 3. Results

### 3.1. Effect of NTs on Senescent HUVECs Morphological Changes

**Figure 1:**
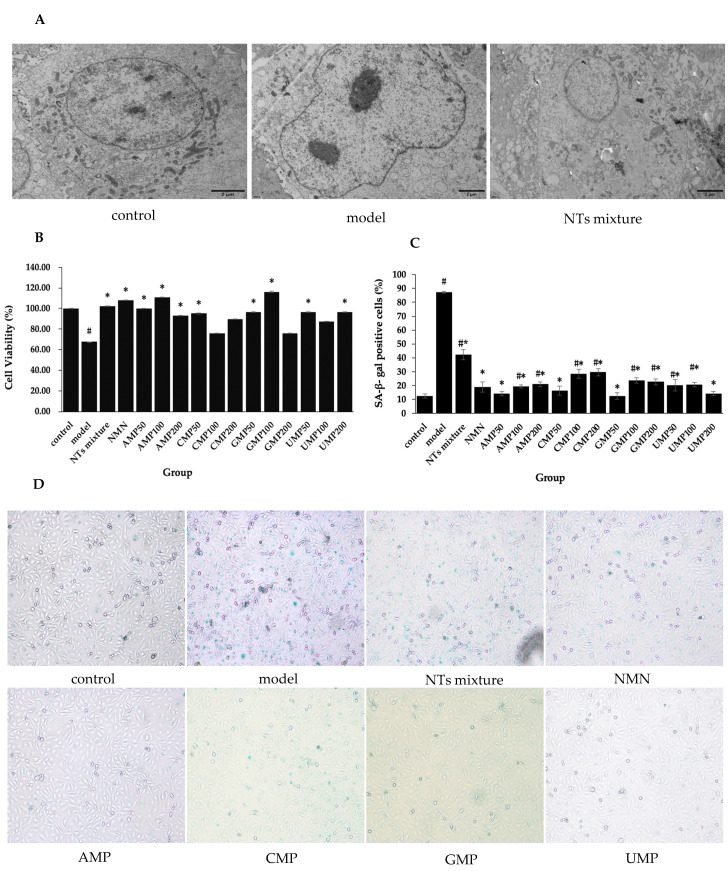
Effect of NTs on senescent HUVECs morphological changes, cell viability, and SA-β-gal activity. (**A**) Effect of NTs on senescent HUVECs morphological changes using transmission electron microscopy (3000×). (**B**) Cell viability evaluation of NTs using the CCK-8 assay (*n* = 5 per group). (**C**) Statistical quantification of SA-β-gal-positive cells (*n* = 3 per group). (**D**) Representative image of SA-β-gal staining. Values represented the mean ± S.D. # *p* < 0.05 versus control group, * *p* < 0.05 versus model group.

Senescent HUVECs displayed an enlarged nucleus, chromatin pyknosis, invaginated nuclear membranes, and a reduced amount of mitochondria. When exposed to 100 µM NTs mixture, the normal nucleus size, homogeneous chromatin, flat nuclear membranes, and relatively major amount of mitochondria was generally maintained in HUVECs (Figure 1A).

### 3.2. Effect of NTs on Senescent HUVECs Viability

Decreased cell viability was observed in senescent HUVECs, while the treatment with NTs leads to considerable improvement (Figure 1B). Compared with the model group, cell viability in the NTs mixture, NMN, AMP50/100/200, CMP50, GMP50/100, and UMP50/200 groups significantly increased. The increased rate relative to the model group was 71.88% (GMP100) > 63.54% (AMP100) > 59.38% (NMN) > 51.04% (NTs mixture) > 47.92% (AMP50) > 42.71% (GMP50/UMP50/UMP200) > 40.63% (CMP50) > 37.50% (AMP200), respectively. From the results above, it is shown that NMN, the NTs mixture, and 4 kinds of NTs were all characterized by enhancing cell viability.

### 3.3. Effect of NTs on SA-β-gal Activity

SA-β-gal is a commonly used senescence marker. Significantly increased SA-β-gal activity was observed in the model group compared with the control group, while the number of SA-β-gal-positive cells decrease after the administration of NMN and NTs compared with the model group (Figure 1C,D). The decreased rate relative to the model group was 85.88% (GMP50) > 83.59% (AMP50/UMP200) > 81.30% (CMP50) > 78.24% (NMN) > 77.87% (AMP100) > 76.72% (UMP50) > 76.34% (UMP100) > 75.95% (AMP200) > 74.04% (GMP200) > 72.90% (GMP100) > 67.56% (CMP100) > 66.03% (CMP200) > 51.53% (NTs mixture). The low dose was more prominent in the AMP, CMP and GMP groups, while the high dose was more effective in the UMP group.

### 3.4. Effect of NTs on Cell Apoptosis in H_2_O_2_-Induced Senescent HUVECs

**Figure 2:**
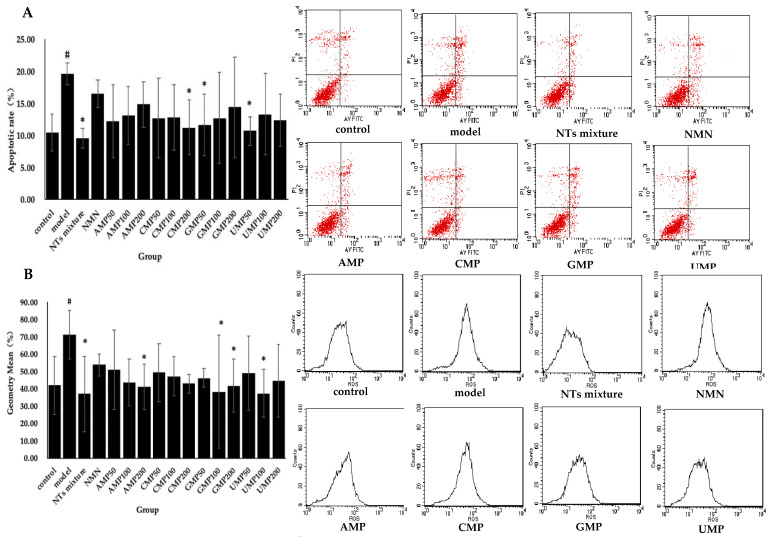

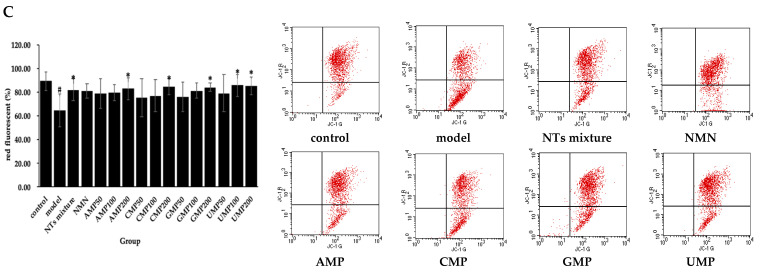
Effect of NTs on senescent HUVECs apoptotic rate, intracellular ROS production, and mitochondrial membrane potential. (**A**) Effect of NTs on senescent HUVECs apoptotic rate; (**B**) Effect of NTs on senescent HUVECs intracellular ROS production; (**C**) Effect of NTs on senescent HUVECs mitochondrial membrane potential. Values represented the mean ± S.D. (*n* = 3 per group). # *p* < 0.05 versus control group, * *p* < 0.05 versus model group.

Significant differences in apoptotic rate were witnessed between the control and model groups. Significantly decreased apoptotic rate was seen in the NTs mixture as well as the CMP200, GMP50, and UMP50 groups (Figure 2A). The decreased rate relative to the model group was 51.16% (NTs mixture) > 45.42% (UMP50) > 42.65% (CMP200) > 40.75% (GMP50) respectively, while no effect on apoptosis rate occurred with the administration of NMN and AMP.

### 3.5. Effect of NTs on Intracellular ROS Production

Enhanced intracellular ROS production in response to the exogenous addition of H_2_O_2_ was observed in the model group compared with the control group, whereas the treatment with NTs significantly decreased intracellular ROS production. As shown in Figure 2B, the AMP200, GMP100/200, UMP100, and the NTs mixture significantly inhibits ROS production, and the inhibition rate relative to the model group was 48.17% (NTs mixture) > 47.60% (UMP100) > 46.14% (GMP100) > 42.35% (AMP200) > 41.45% (GMP200), respectively. No effect on ROS production occurred with the administration of NMN and CMP.

### 3.6. Effect of NTs on Senescent HUVECs Mitochondrial Membrane Potential (JC-1)

The exogenous addition of H_2_O_2_ induced mitochondrial membrane potential depolarization in HUVECs. Meanwhile, compared with the model group, a positive effect of NTs mixture, AMP200, CMP200, GMP200, and UMP50/100 on mitochondrial membrane potential was observed (Figure 2C). The increased rate of red fluorescent relative to the model group was 33.23% (UMP100) > 32.43% (UMP200) > 30.91% (CMP200) > 30.38% (GMP200) > 28.90% (AMP200) > 27.34% (NTs mixture), respectively.

### 3.7. Effect of NTs on Senescent HUVECs SASP

**Figure 3:**
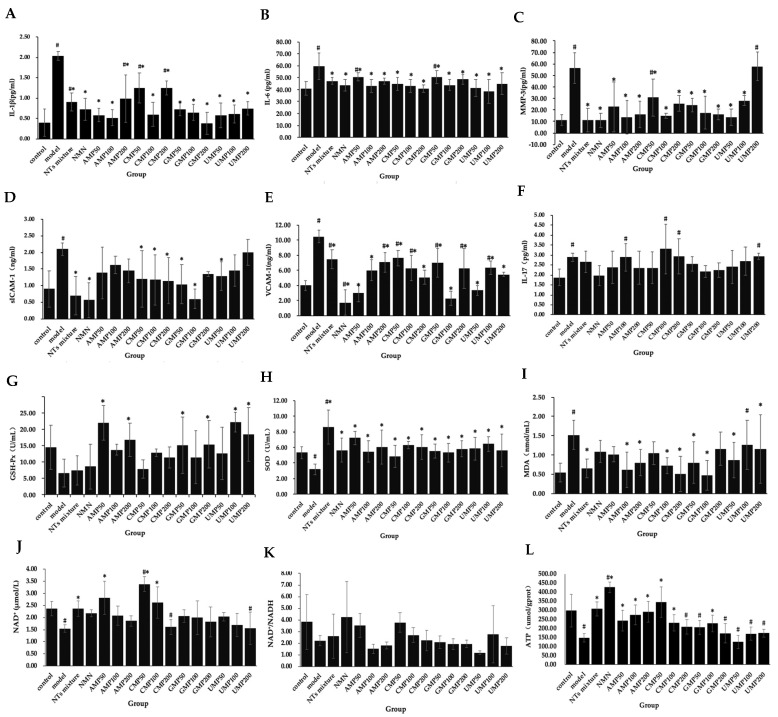
Effect of NTs on senescent HUVECs SASP, antioxidant activity, NAD^+^ levels, NAD^+^/NADH ratio, and ATP production. (**A**) IL-1β concentration in senescent HUVECs supernatant; (**B**) IL-6 concentration in senescent HUVECs supernatant; (**C**) MMP-3 concentration in senescent HUVECs supernatant; (**D**) sICAM-1 concentration in senescent HUVECs supernatant; (**E**) VCAM-1 concentration in senescent HUVECs supernatant; (**F**) IL-17 concentration in senescent HUVECs supernatant; (**G**) GSH-Px activities in senescent HUVECs; (**H**) SOD activities in senescent HUVECs; (**I**) MDA levels in senescent HUVECs; (**J**) NAD^+^ levels in senescent HUVECs; (**K**) NAD^+^/NADH in senescent HUVECs; (**L**) ATP production in senescent HUVECs. Values represented the mean ± S.D. (*n* = 3 per group). # *p* < 0.05 versus control group, * *p* < 0.05 versus model group.

To evaluate the effect of NTs on the senescent HUVECs inflammatory phenotype, the secretion of IL-6, IL-1β, IL-17, MMP-3, ICAM-1, and VCAM-1 was measured. As shown in Figure 3A–F, compared with the control group, the concentrations of IL-1β, IL-6, IL-17, ICAM-1, VCAM-1, and MMP-3 in the model group significantly increased, indicating that the H_2_O_2_-induced senescent HUVECs were in an inflammatory state.

The secretion of IL-1β significantly decreased in the NTs mixture, NMN, and all of the AMP, CMP, GMP, and UMP groups (Figure 3A); the decreased rate relative to the model group was 81.26% (GMP200) > 74.41% (AMP100) > 71.54% (UMP50) > 71.19% (AMP50) > 70.41% (CMP100) > 70.28% (UMP100) > 68.16% (GMP100) > 64.30% (NMN) > 64.08% (GMP50) > 63.12% (UMP200) > 55.37% (NTs) > 51.32% (AMP200) > 38.72% (CMP50) > 38.51% (CMP200), respectively.

The secretion of IL-6 significantly decreased in the NTs mixture, NMN, and all of the AMP, CMP, GMP, and UMP groups (Figure 3B); the decreased rate relative to the model group was 35.60% (UMP100) > 31.67% (CMP200) > 30.99% (UMP50) > 27.81% (AMP100) > 27.72% (CMP100) > 26.70% (NMN) > 26.69% (GMP100) > 25.25% (CMP50) > 24.78% (UMP200) > 21.22% (AMP200) > 20.87% (NTs mixture) > 18.81% (GMP200) > 15.67% (GMP50) > 15.36% (AMP50), respectively.

Compared with the model group, the MMP-3 levels in the NTs mixture, NMN, AMP50/100/200, CMP50/100/200, GMP50/100/200, and UMP50/100 groups significantly decreased (Figure 3C); the decreased rate relative to the model group was 80.62% (NTs mixture) > 80.48% (NMN) > 75.99% (AMP100) > 75.76% (UMP50) > 73.32% (CMP100) > 71.11% (AMP200) > 70.99% (GMP200) > 68.85% (GMP100) > 59.79% (AMP50) > 57.02% (GMP50) > 54.70% (CMP200) > 50.07% (UMP100) > 45.60% (CMP50), respectively.

The secretion of sICAM-1 in the NTs mixture, NMN, CMP50/100/200, GMP50/100, and UMP50 groups significantly decreased (Figure 3D), the decreased rate relative to the model group was 73.13% (NMN) > 71.52% (GMP100) > 66.64% (NTs mixture) > 50.41% (GMP50) > 45.53% (CMP200) > 43.90% (CMP100) > 42.27% (CMP50) > 39.05% (UMP50), respectively.

The secretion of VCAM-1 significantly decreased in the NTs mixture, NMN, and all of the AMP, CMP, GMP, and UMP groups (Figure 3E); the decreased rate relative to the model group was 83.56% (NMN) > 78.30% (GMP100) > 71.13% (AMP50) > 68.26% (UMP50) > 51.63% (CMP200) > 48.09% (UMP200) > 42.64% (AMP100) > 40.06% (GMP200) > 39.96% (CMP100) > 39.39% (UMP100) > 33.27% (GMP50) > 32.70% (AMP200) > 28.78% (NTs mixture) > 27.34% (CMP50), respectively.

No effect on IL-17 occurred after the administration of NMN and NTs (Figure 3F).

### 3.8. Effect of NTs on H_2_O_2_-Induced Decreased Antioxidant Activity in HUVECs

H_2_O_2_-induced senescent HUVECs display significantly decreased the activities of SOD and notably increased MDA levels. GSH-Px activities were not significantly altered in senescent HUVECs in the presented experiment.

GSH-Px activities in the AMP50/100, GMP50/200, UMP100/200 groups significantly increased more than the model group (Figure 3G); the increased rate relative to the model group was 239.45% (UMP100) > 235.78% (AMP50) > 182.26% (UMP200) > 157.19% (AMP200) > 135.78% (GMP200) > 132.26% (GMP50), respectively.

Compared with the model group, SOD activities in NTs, NMN CMP100/200, and all of the AMP/GMP/UMP groups significantly increased (Figure 3H), the increased rate relative to the model group was 170.13% (NTs mixture) > 127.36% (AMP50) > 102.20% (UMP100) > 96.86% (CMP100) > 89.94% (AMP200/CMP200) > 83.33% (UMP50) > 82.70% (GMP200) > 77.36% (NMN) > 77.04% (UMP200) > 72.96% (GMP50) > 71.70% (AMP100) > 67.61% (GMP100), respectively.

Compared with the model group, MDA levels in the NTs, AMP100/200, CMP100/200, GMP50/100, and UMP50/200 groups significantly decreased (Figure 3I), the decreased rate relative to the model group was 69.08% (GMP100) > 66.45% (CMP200) > 59.21% (AMP100) > 57.24% (NTs mixture) > 52.63% (CMP100) > 47.37% (AMP200/GMP50) > 42.76% (UMP50), respectively.

### 3.9. Effect of NTs on NAD^+^ Levels and NAD^+^/NADH

As shown in Figure 3J,K, NAD^+^/NADH levels in the control group and senescent HUVECs did not differ, but NAD^+^ levels significantly decreased in senescent HUVECs compared with the control group. While the NTs mixture, AMP50, and CMP50/100 significantly increased NAD^+^ levels, the increased rate relative to the model group was 118.71% (CMP50) > 81.56% (AMP50) > 69.42% (CMP100) > 52.60% (NTs mixture), respectively.

### 3.10. Effect of NTs on Senescent HUVECs ATP Production

Decreased ATP production in response to H_2_O_2_-induced HUVECs senescence was observed compared with the control group. A significantly higher ATP production was seen in the NTs mixture, NMN, AMP50/100/200, CMP50/100, and GMP100 groups (Figure 3L), the increased rate relative to the model group was 190.80% (NMN) > 134.74% (CMP50) > 109.52% (NTs mixture) > 98.29% (AMP200) > 64.95% (AMP50) > 57.01% (CMP100) > 54.82% (GMP100), respectively.

### 3.11. Effect of NTs on the Protein Expression of p16^INK4A^, p21^Waf1/Cip1^, p15^INK4b^, and ACE-2

**Figure 4:**
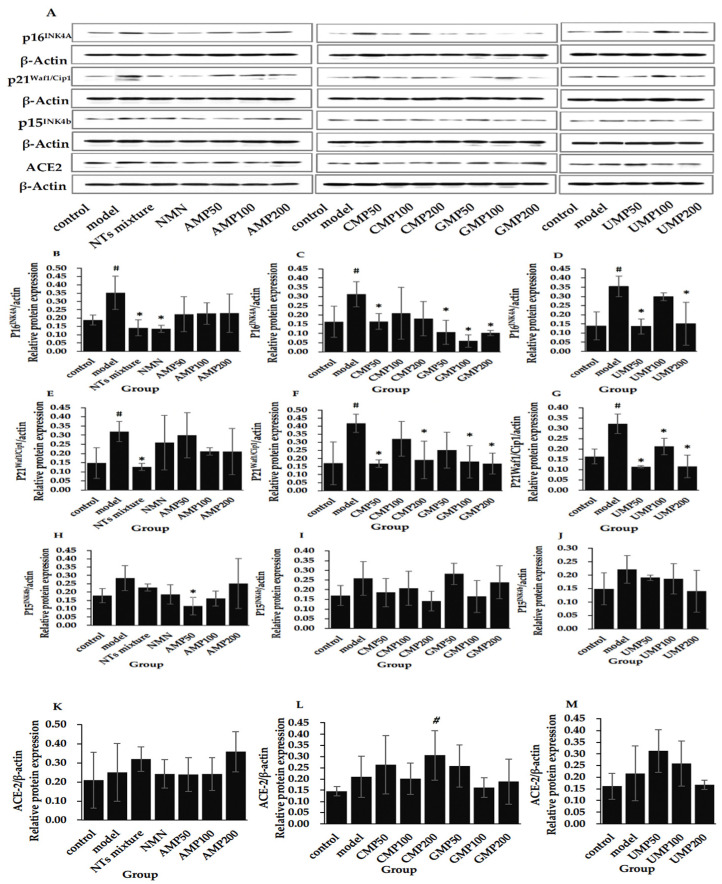
Effect of NTs on the protein expression of p16^INK4A^, p21^Waf1/Cip1^, p15^INK4b^, and ACE-2. (**A**) The Western blot strip in all groups; (**B**,**E**,**H**,**K**). The protein expression of p16^INK4A^, p21^Waf1/Cip1^, p15 ^INK4b^, and ACE-2 in the NMN and AMP50/100/200 groups; (**C**,**F**,**I**,**L**). The protein expression of p16^INK4A^, p21^Waf1/Cip1^, p15 ^INK4b^, and ACE-2 in the CMP50/100/200 and GMP50/100/200 groups; (**D**,**G**,**J**,**M**). The protein expression of p16^INK4A^, p21^Waf1/Cip1^, p15 ^INK4b^, and ACE-2 in the UMP50/100/200 group. Values represented the mean ± S.D. (*n* = 3 per group). # *p* < 0.05 versus control group, * *p* < 0.05 versus model group.

Figure 4 shows that the expression of senescence marker proteins p16^INK4A^ and p21^Waf1/Cip1^ significantly increased in the model group compared with the control group.

The treatment of the NTs mixture, NMN, CMP50, GMP50/100/200, and UMP50/200 significantly downregulated the expression of p16^INK4A^ (Figure 4B–D), the decreased rate relative to the model group was 80.99% (CMP100) > 67.09% (GMP200) > 65.98% (GMP50) > 61.80% (UMP50) > 61.54% (NMN) > 59.80% (NTs mixture) > 57.56% (UMP200) > 47.42% (CMP50), respectively.

Furthermore, the administration of the NTs mixture, CMP50/CMP200, GMP100/200, and UMP50/100/200 is able to inhibit the protein expression of the p21^Waf1/Cip1^ (Figure 4E–G); the decreased rate relative to the model group was 64.50% (UMP50) > 64.30% (UMP200) > 60.47% (NTs mixture) > 59.71% (GMP200) > 59.64% (CMP50) > 56.75% (GMP100) > 54.33% (CMP200) > 34.15% (UMP100), respectively.

Although there was a tendency toward higher expression, the expression of p15^INK4b^ was not significantly altered in senescent HUVECs compared with the control group, and a significantly decreased level of p15^INK4b^ was seen in the AMP50 group compared with the model group (Figure 4H–J). No difference in ACE-2 expression was witnessed between the control and model groups, and it was significantly increased in the CMP200 group compared with the control group (Figure 4K–M).

## 4. Discussion

Currently, CVDs account for approximately 39.6% of age-related diseases [29], and CVDs-induced insufficient blood supply may further accelerate the aging process. By this, we investigated the role of NTs in attenuating HUVECs senescence. The free radical theory of aging proposes that the accumulation of molecular oxidative damage increases as the organism ages and is postulated to be a major causal factor of senescence [30]. Although recent data support a role for ROS in the activation of compensatory homeostatic responses, ROS aggravate age-associated damage at certain higher doses [31]. Multiple studies have successfully built a senescence model by aggravating oxidative stress [32,33]. Based on this factual framework, we have established the model of H_2_0_2-_induced senescence.

We assessed several senescence markers during the experiment, and our data showed that exposure to 200 µM H_2_O_2_ for 4 h significantly upregulated p16^INK4A^, p21^Waf1/Cip1^, and SA-β-gal expression in HUVECs, confirming that the senescence model was well-established. Given a permanent-growth arrest coupled to acute oxidative stress, reduced cell viability and increased apoptosis rate were observed in senescent HUVECs. However, our data revealed that the expression of p15^INK4b^, a cyclin-dependent kinase inhibitor that mediates cell cycle arrest [34], and ACE-2, a hallmark of cardiopulmonary aging [35], were not affected in the present study.

We show that NTs retards oxidative stress-induced HUVECs senescence. The expression of p16^INK4A^, p21^Waf1/Cip1^, and SA-β-gal significantly reduced in the NTs treated group. Correspondently, cell viability and apoptosis rate returned to the normal levels. Further analysis of the data supported the comprehensively positive role of the NTs mixture, CMP50, GMP100, and UMP50/100 in delaying HUVECs senescence, and GMP100 was the most excellent candidate (Figure S2A,B). While NMN specifically downregulated the expression of p16^INK4A^, it did not affect the p21^Waf1/Cip1^ (Figure S2B). AMP significantly augmented cell viability and suppressed SA-β-gal activity in HUVECs at low and middle doses, but they both demonstrated an incapacity for lowering p16^INK4A^ and p21^Waf1/Cip1^ expression (Figure S2C). Interestingly, AMP50 significantly downregulated p15^INK4b^ expression compared to the model group. Given that cellular senescence was identified as a hallmark of aging, the therapeutic strategies targeting senescent cells may attenuate age-associated pathologies and prolong lifespan. Our data revealed the role of NTs in delaying HUVECs senescence, thus revealing its potential application for CVDs therapy. Our finding is partially consistent with that of Xu, who also found that elevated intracellular adenosine promoted proliferation in human endothelial cells [36]. Furthermore, previous studies in our lab also found that the long-term feeding of NTs prolonged the average and maximum lifespan of SD rats and reduced the death rate due to tumors [25].

According to recent studies, SASP, the hallmarks of cellular senescence [37], can drive age-related pathologies through paracrine and likely endocrine effects [38]. Along this line, the suppression of SASP is rational for retarding senescence and attenuating age-related pathologies. Our data showed that NTs have strong anti-inflammatory properties in HUVECs, and the response intensity of each group was relatively consistent, especially to IL-6, IL-1β. The NTs mixture, NMN, CMP50/100/200, GMP50/100, and the UMP50 groups significantly decreased all of the five indexes, while NMN and GMP100 exhibited the greatest performance (Figure S3A,B). AMP did not affect sICAM-1 secretion, but it was highly active in inhibiting IL-6, IL-1β, VCAM-1, and MMP-3 secretion, especially at a low dose (Figure S3C). For UMP, the low dose was also more effective (Figure S3F), while in GMP and CMP, the middle dose showed greater performance (Figure S3D,E). Furthermore, IL-17 was pinpointed as an aging marker elevated in the aged lung, heart, and vessel [35]. Consistent with this, our data showed that IL-17 secretion was upregulated in senescent HUVECs, but NTs did not alter the upregulated state in the present study. Meanwhile, NTs are essential for maintaining the optimal immune response, and several previous studies supported their anti-inflammatory action [18].

Given that oxidative stress aggravates age-associated damage, the alleviation of oxidative damage or enhancement of antioxidant capacity by NTs treatment is a potential mechanism for retarding senescence in HUVECs. We showed that NTs exhibit superior antioxidant properties through different mechanisms (Figure S4A). Only AMP200 significantly altered overall the four indexes, indicating the integrative competencies of AMP200 to suppress oxidative damage. GMP100 and CMP200 were more inclined to eliminate ROS and MDA (Figure S4B), while AMP50 and UMP100 were inclined to increase GSH-Px and SOD activities (Figure S4C). In the present study, the GHS-Px activity in the model group tended to decrease, but there was no difference. Therefore, if we ignore the effect of NTs on GSH-Px (Figure S4D), the performance of NTs mixture was outstanding and all-sided, followed by AMP200 and GMP100 (Figure S4E). In addition, our previous studies have demonstrated that NTs inhibited the decrease in antioxidant enzyme activity and the increase in lipid peroxidation product in aged animal models [25].

Mitochondrial dysfunction occurs with cells and as organisms age, thus resulting in the loss of mitochondrial membrane potential and reduced ATP production. In turn, this opens up the possibility of retarding HUVECS senescence by improving mitochondrial function. In the present study, mitochondrial function was estimated by mitochondrial membrane potential and the ability of ATP production. From the data in Figure S5, it is apparent that the NTs mixture and a high dose of NTs exerted a protective effect on the mitochondrial membrane, while for ATP production, the effects of AMP and CMP were generally superior to those of GMP and UMP. The NTs mixture and AMP200 significantly increased both mitochondrial membrane potential and ATP production. There was a complex link between NAD^+^ metabolism and mitochondrial function, and decreased cellular NAD^+^ concentrations occur during aging [39]. Thus, we involved the NAD^+^ level in our test, and the effects of the NTs mixture, AMP, and CMP were also highlighted. We supposed that the senescence ameliorative effect of NTs on HUVECs may depend on NAD^+^ sensing by Sirt1. Increased levels of NAD^+^ enhance Sirt1 activity and lead to the activating deacetylation of PGC-1α and increased mitochondrial biogenesis and function [40]. Sirt1 is also linked to aging by regulating inflammation through NF-κB signaling [39]. Meanwhile, this study broadly supports the work of other studies in animal models, which showed that exogenous NTs could increase oxidative phosphorylation [20], enhance the repair of damaged mitochondrial DNA [41], and promote the early recovery of mitochondrial function changes after stress [28]. However, this study has been unable to demonstrate that NTs enhance the turnover between oxidized and reduced forms of NAD^+^, as described previously [20].

NMN is a key NAD^+^ intermediate; extensive studies have shown that supplementing NMN ameliorates age-associated pathophysiologies and disease conditions, including CVDs [42]. NMN is also a derivative of NTs and has a similar chemical structure, so we used the NMN administration group as a positive control for the present study and evaluated its effect on senescent HUVECs. We showed that NMN retards H_2_O_2_-induced HUVECs senescence through downregulating the p16^INK4A^ and SASP, promoting cell viability and ATP production. However, NMN has a weak effect on NAD^+^ in this investigation compared to those of other studies [43,44]. The antioxidant and protecting mitochondrial function of NTs is superior to NMN in the present study.

Overall, the present study has supported the free radical theory of aging, confirming that aggravated oxidative stress triggers HUVECs senescence. These findings, while preliminary, provide the first evidence for the role of NTs in retarding HUVECs senescence, and the mechanism may relate to its anti-inflammatory, anti-oxidant activities and the effect of improving mitochondrial functions. The findings of this investigation complement those of earlier studies; we comprehensively assessed the anti-inflammatory, anti-oxidant, and protecting mitochondria activities of four kinds of NTs and their mixtures. The comprehensive analysis of NTs undertaken here has extended our knowledge of the functional characteristics, the effective dose of different purine and pyrimidine NTs, as well as its optimum proportion. This new understanding should help expand the research fields of NTs widely.

There were several limitations in our study. First, to satisfy the overwhelming cell demand of the multiple test groups, we chose the cell line in our research. Disappointingly, the H_2_O_2_-induced premature senescent model cannot fully simulate the natural cellular senescence process. The study should be repeated using replicative senescent cells or primary cultured senescent endothelial cells. Second, because of the heavy workload and long literary piece, the present study only focuses on functional assessment; the fundamental mechanisms responsible for the retarding HUVECs senescence effect of NTs have not yet been fully elucidated. Further research should be undertaken to explore how NTs are absorbed by HUVECs and their molecular mechanisms of delaying cellular senescence. Considerably more work will need to be done to determine the role of NTs in CVDs treatment, including in vivo experiments and large randomized controlled trials. Notwithstanding these limitations, the study suggests that NTs can restore the functional changes during the HUVECs senescence process and provide a theoretical foundation for future research.

## 5. Conclusions

In conclusion, we have shown that NTs retard the oxidative stress-induced senescence of HUVECs, decrease the expression of senescence markers, promote cell viability, augment antioxidant activity, ameliorate SASP and mitochondrial dysfunction, and increase NAD^+^ production during the HUVECs senescence process. GMP100 was most excellent in delaying HUVECs senescence, while the effect of the NTs mixture was full-scaled. These results stated that the mechanisms of the amelioration of HUVECs senescence actions of NTs could be related to their antioxidant and anti-inflammation properties as well as their ability to promote cell proliferation and protect mitochondrial function activities.

## Data Availability

The data presented in this study are available on request from the corresponding author. The data are not publicly available due to privacy.

## Supplementary Materials

The following are available online at https://www.mdpi.com/article/10.3390/nu13093279/s1, Figure S1: H_2_O_2_-induced senescence in HUVECs, Figure S2: Comprehensive analysis of NTs on retarding HUVECs senescence, Figure S3: Comprehensive analysis of NTs on inhibiting SASP in HUVECs, Figure S4: Comprehensive analysis of NTs on alleviating oxidative damage in HUVECs, Figure S5. Comprehensive analysis of NTs on mitochondrial function in HUVECs.
