# Supplementary material for: Dietary Nucleotides Retard Oxidative Stress-Induced Senescence of Human Umbilical Vein Endothelial Cells
Source: Nutrients. 2021 Sep 20;13(9):3279. doi: 10.3390/nu13093279 (PMC8472005; doi:10.3390/nu13093279)
Supplement: Supplementary file 1 [file nutrients-13-03279-s001.zip › nutrients-1348363-SI.pdf]

Treated HUVECs with 0, 50, 100, 200, 400, and 800 µM H_2O_2 for 4h and for the estimation of cellular senescence, cell viability, SA-β-gal activity, and $p16^{INK4A}$ and $p21^{Waf1/Cip1}$ protein expression were evaluated. The concentration of H_2O_2, which resulted in a significant decrease in cell viability without severe cytotoxicity and a significant increase in SA-β-gal activity and expression of $p16^{INK4A}$ and $p21^{Waf1/Cip1}$ protein, was selected for subsequent study (Figure S1).

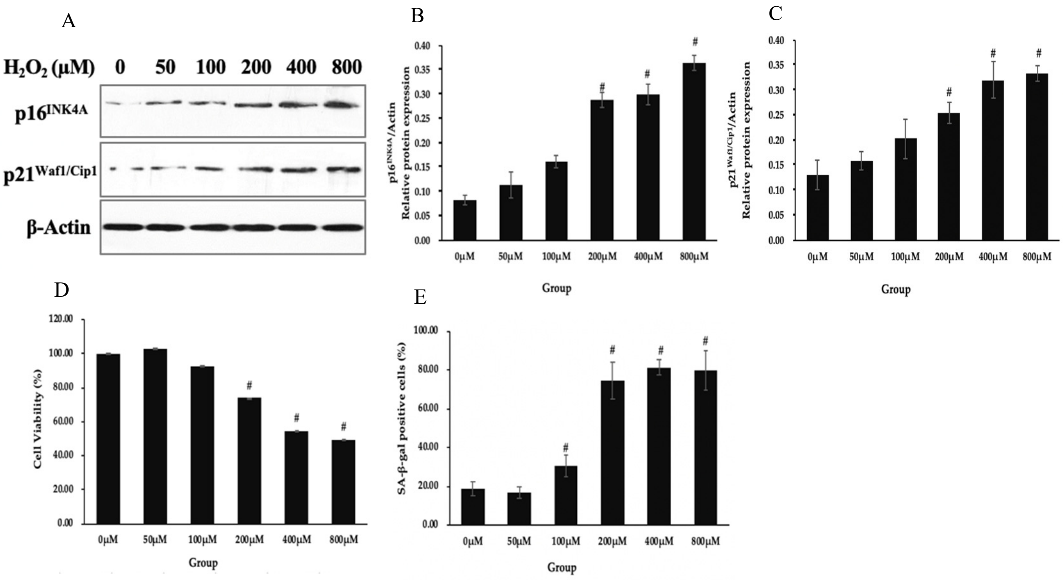

Figure S1. H_2O_2-induced senescence in HUVECs. A. The western blot strips in all groups. B. The effect of H_2O_2 on the protein expression of $p16^{INK4A}$ in HUVECs. C. The effect of H_2O_2 on the protein expression of $p21^{Waf1/Cip1}$ in HUVECs. D. The effect of H_2O_2 on the cell viability in HUVECs. E. The effect of H_2O_2 on the SA-β-gal activity in HUVECs.

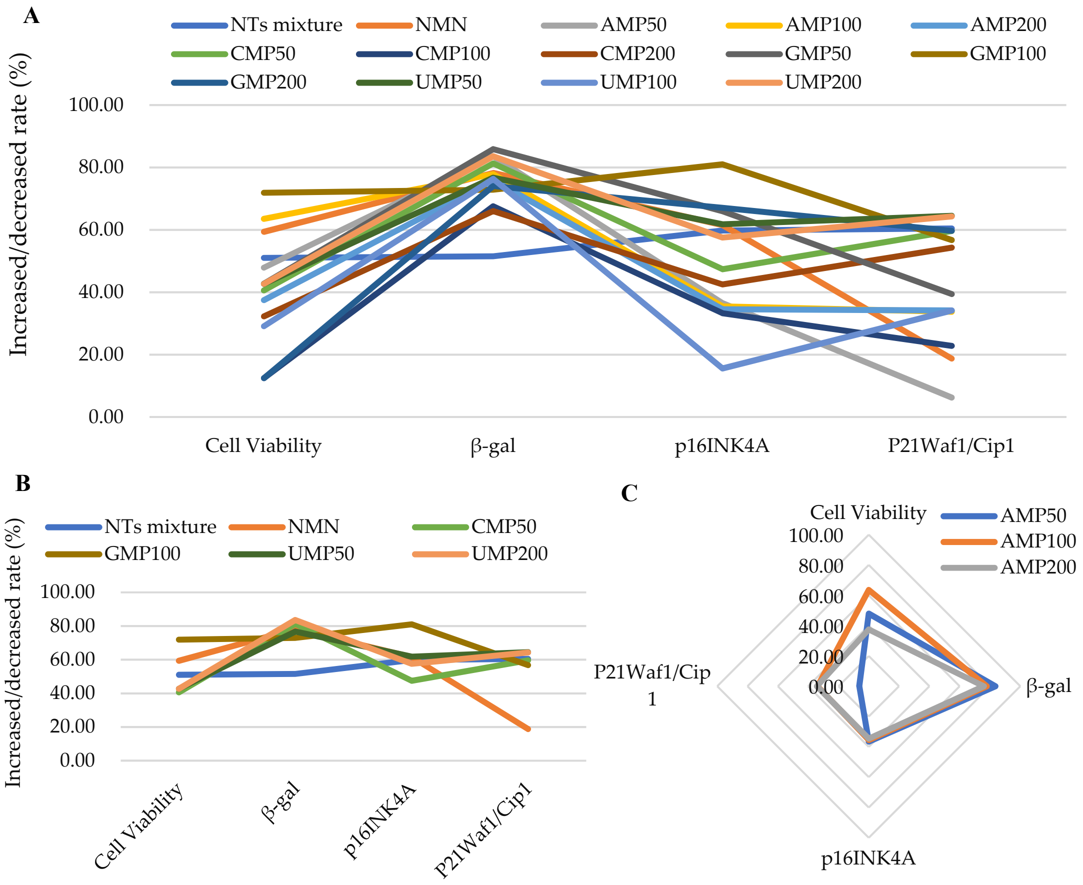

Figure S2. Comprehensive analysis of NTs on retarding HUVECs senescence. A. The overall trend of NTs on retarding HUVECs senescence; B. Selected groups with great performance on retarding HUVECs senescence; C. The overall trend of AMP on retarding HUVECs senescence. Values represented the decreased/ increased rate relative to model group, decreased rate = (model group – treated group)/ model group, increased rate = (treated group – model group)/ model group.

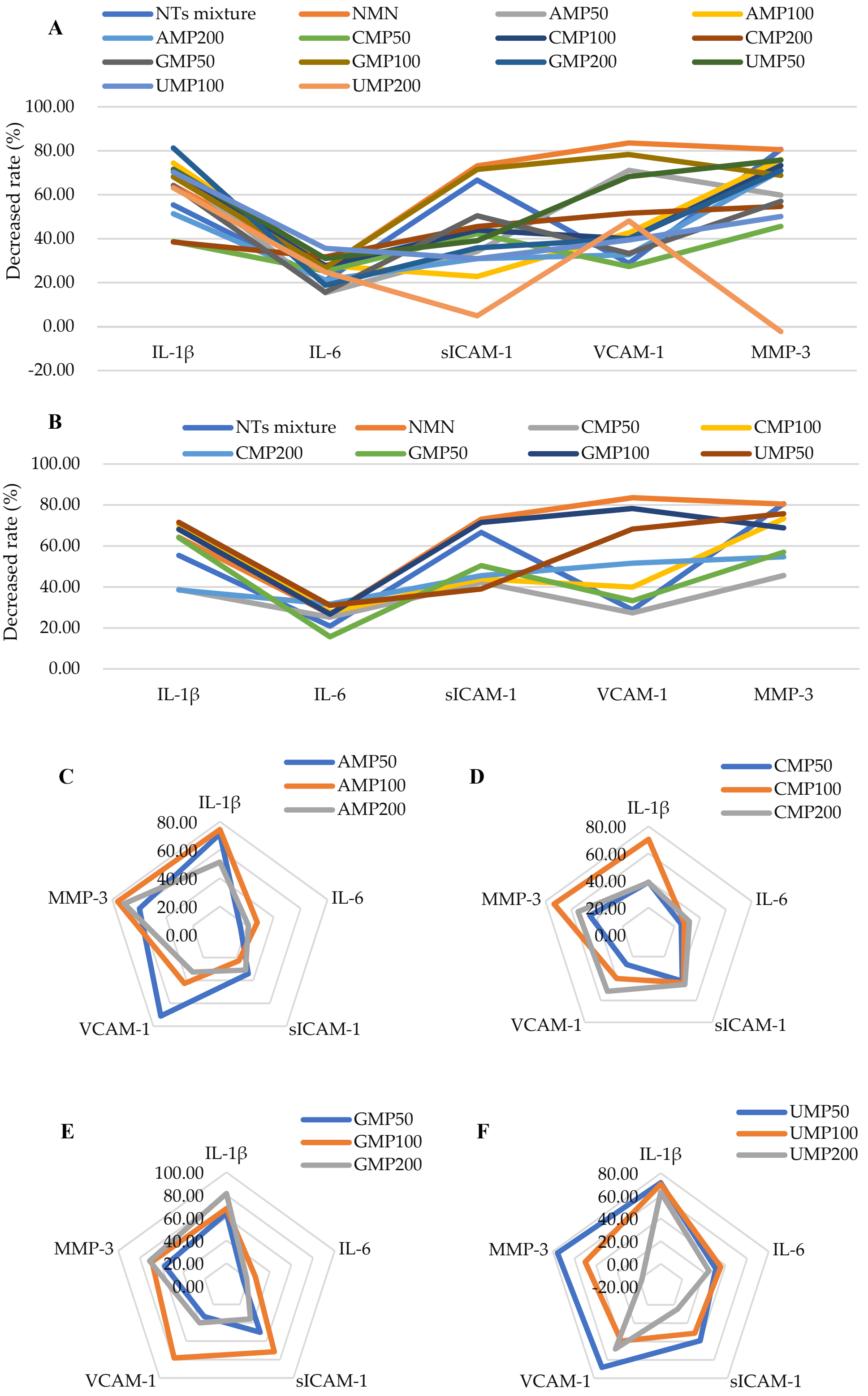

Figure S3. Comprehensive analysis of NTs on inhibiting SASP in HUVECs. A. The overall trend of NTs on inhibiting SASP in HUVECs; B. The overall trend of selected groups which significantly decreased all of 5 index; C. The overall trend of AMP on inhibiting SASP in HUVECs. D. The overall trend of CMP on inhibiting SASP in HUVECs. E. The overall trend of GMP on inhibiting

SASP in HUVECs. F. The overall trend of UMP on inhibiting SASP in HUVECs. Values represented the decreased rate relative to model group, decreased rate = (model group – treated group)/ model group.

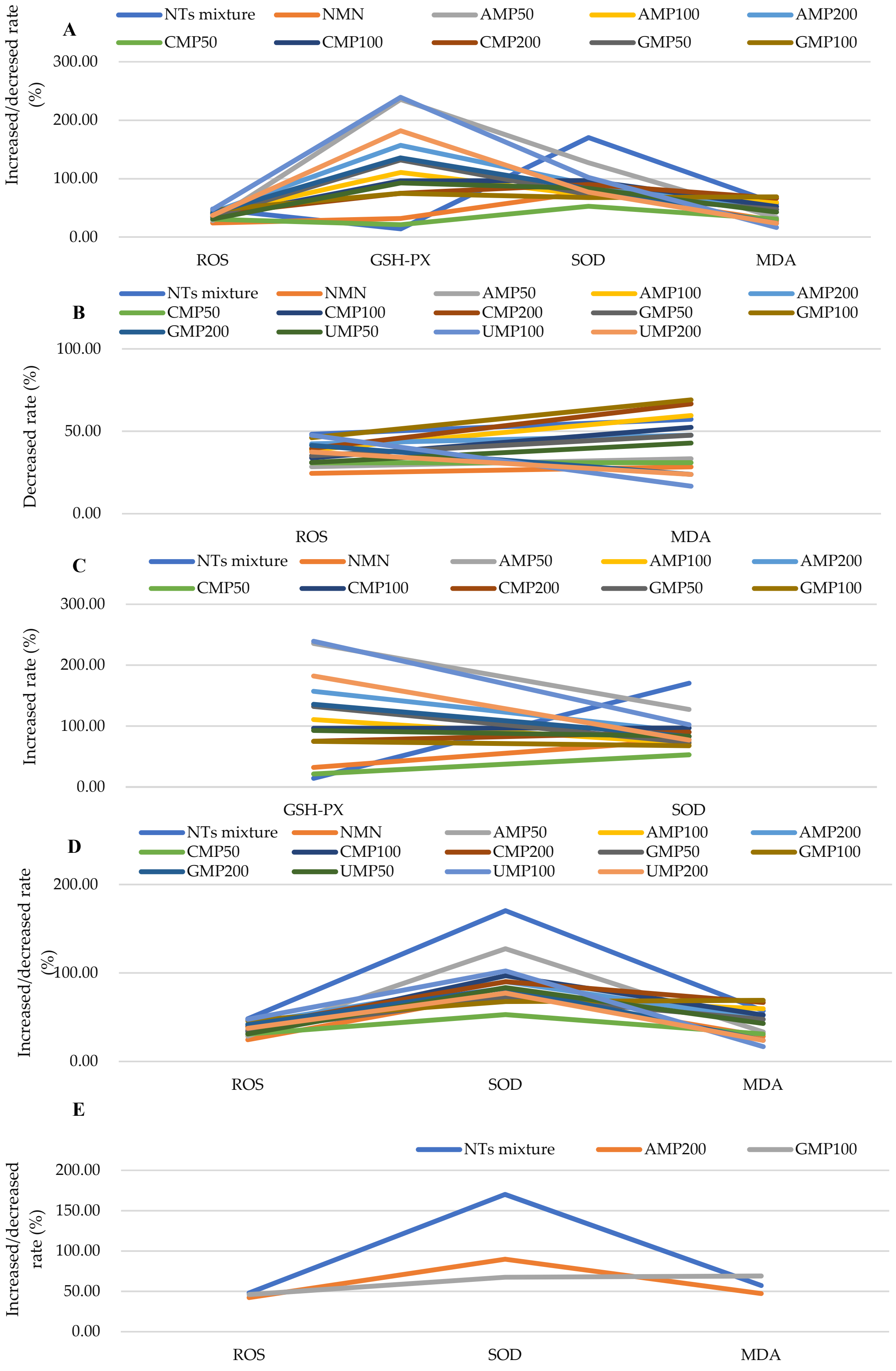

Figure S4. Comprehensive analysis of NTs on alleviating oxidative damage in HUVECs. A. The overall trend of NTs on alleviating oxidative damage in HUVECs; B. The overall trend of NTs on eliminating ROS and MDA; C. The overall trend of NTs on increasing GSH-Px and SOD activities. D. The overall effect of NTs on ROS, SOD and MDA in HUVECs. E. The overall trend of selected groups which significantly altered ROS, SOD and MDA in HUVECs. Values represented the decreased/ increased rate relative to model group, decreased rate = (model group – treated group)/ model group, increased rate = (treated group – model group)/ model group.

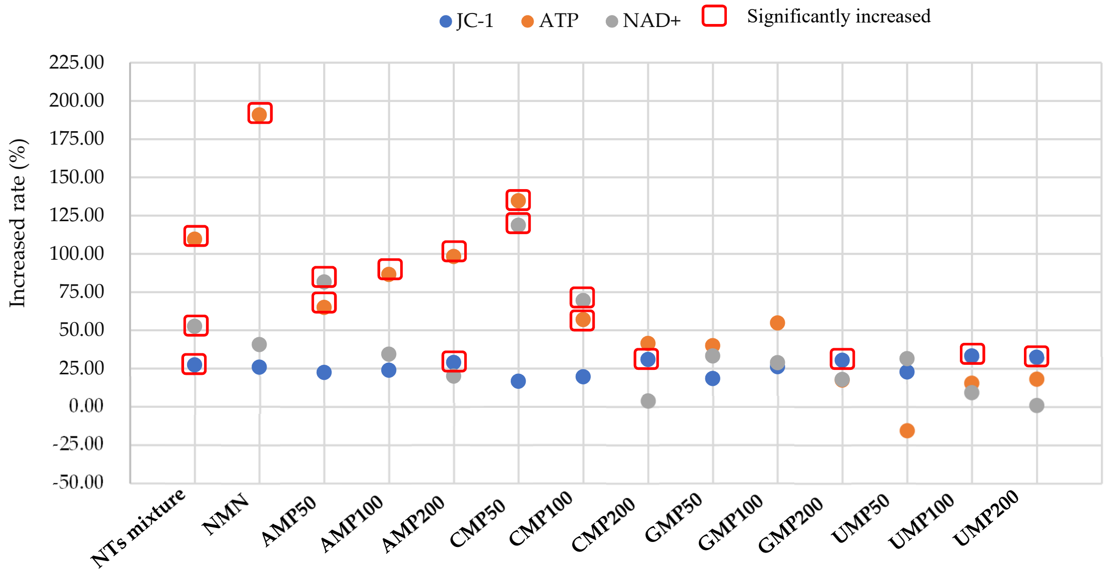

Figure S5. Comprehensive analysis of NTs on mitochondrial function in HUVECs. Values represented the increased rate relative to model group, increased rate = (treated group – model group)/ model group.
